# Obligations to Report Outbreaks of Foodborne Disease under the International Health Regulations (2005)

**DOI:** 10.3201/eid1409.080468

**Published:** 2008-09

**Authors:** Martyn Kirk, Jennie Musto, Joy Gregory, Kathleen Fullerton

**Affiliations:** Australian Government Department of Health & Ageing, Canberra, Australian Capital Territory, Australia (M. Kirk, K. Fullerton); Australian National University, Canberra (M. Kirk); New South Wales Department of Health, Sydney, New South Wales, Australia (J. Musto); Department of Human Services, Melbourne, Victoria, Australia (J. Gregory)

**Keywords:** outbreak, foodborne, international, International Health Regulations, dispatch

## Abstract

Every year, Australia identifies 2–3 outbreaks associated with imported foods. To examine national authorities’ obligations under the International Health Regulations (2005), we reviewed outbreaks in 2001–2007 that implicated internationally distributed foods. Under these regulations, 6 (43%) of 14 outbreaks would have required notification to the World Health Organization.

During the past 2 decades the global trade in food has increased, making outbreaks associated with internationally distributed foods more common (*1*). These outbreaks are challenging to identify and control. This is despite the involvement of multinational agencies, such as the World Health Organization (WHO), the European Centre for Disease Prevention and Control, and surveillance networks, such as PulseNet International and the European Foodborne Viruses Network, which use molecular techniques to rapidly compare infecting strains (*1*–*4*). Food is a silent vehicle for spreading pathogens and chemicals across country borders (*5*). Whenever agencies responsible for health, agriculture, or food safety identify contaminated foods that are imported or exported, the potential for human illness to occur in other countries exists.

WHO recently revised the legally binding International Health Regulations (IHR) to respond more effectively to the increasing spread of disease internationally (*6*). IHR (2005) are based on a risk assessment approach and came into force on June 15, 2007. Under IHR (2005), countries are required to designate or establish a National IHR Focal Point, which should be a national center for urgent communications under the regulations. These regulations include a decision-making instrument that lead National Focal Points through a series of 4 questions to assist them in making a decision to report events to WHO for international alert and response (Figure) (*7*). IHR (2005) cover events of international importance that involve contaminated food and outbreaks of foodborne disease. In 2004, WHO, in collaboration with the Food and Agriculture Organization of the United Nations, launched the International Food Safety Authorities Network (INFOSAN) to improve food safety information exchange and cooperation, including a food safety emergency component (INFOSAN Emergency; see www.who.int/foodsafety/fs_management/infosan/en). WHO has developed guidance to illustrate how INFOSAN Emergency complements processes under IHR (2005) (*8*).

**Figure:**
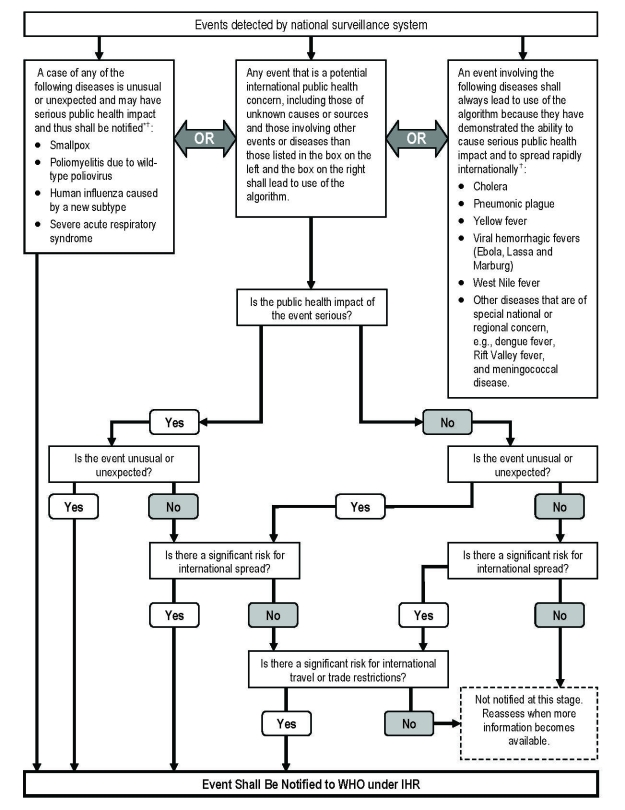
Decision instrument for the assessment and notification of events that may constitute a public health emergency of international concern under International Health Regulations (IHR) (2005). WHO, World Health Organization. *As per WHO case definitions. †The disease list shall be used only for the purposes of these regulations; adapted from Annex II of IHR (2005), reported in (*7*).

In Australia, health departments in 6 states and 2 territories led multiagency teams to investigate and control ≈100 outbreaks of foodborne disease that affected 2,000–4,000 people each year. The results of these investigations are summarized and reported to OzFoodNet, Australia’s national system of foodborne disease surveillance. When outbreaks are spread across jurisdictional or country borders, OzFoodNet coordinates national responses to determine the cause of the outbreak and prevent further spread.

In this report, we assess well-characterized outbreaks of foodborne disease traced to internationally distributed foods. We used questions in the IHR decision-making algorithm to examine whether these outbreaks would be potentially reportable under IHR (2005). We also make some observations on investigating and managing these international outbreaks from recent experiences, including interaction with INFOSAN Emergency.

## The Outbreaks

In the 7-year period 2001–2007, 14 (1.8%) of 768 foodborne outbreaks were associated with foods that were distributed internationally (Appendix Table). In total, these outbreaks affected at least 542 persons in Australia, 4.4% (542/12,423) of all those affected by foodborne disease outbreaks during the period. The median size of these outbreaks was 20 persons (range 3–230). The number of persons affected in other countries as a result of these events was unknown. Given the nature of foodborne disease, more outbreaks that we were unable to identify were likely associated with internationally distributed foods.

Several point-source outbreaks were related to each other by a common food source, even though the foods were often branded differently and supplied by different companies. The outbreaks of suspected norovirus infection (outbreaks 4, 6, and 9) were associated with individually quick frozen (IQF) oysters all harvested from the same region in Japan; this association was later confirmed after a national investigation into 3 related outbreaks (outbreaks 7, 8, and 10) (*9*). These outbreaks occurred over a 3-year period and resulted in Australia’s imposing restrictions on importation of IQF oysters from this growing area.

No outbreaks was considered to be of “serious public health impact” because of their small size and moderate severity. In 4 (29%) of 14 outbreaks, the event was considered “unusual or unexpected” in Australia because of novel disease-causing agents. However, agents considered novel in Australia were common causes of disease in the country exporting the food (*10*). In 5 (36%) of the 14 outbreaks, food had been distributed to other countries, resulting in multinational food recalls; 4 more events had the potential to spread to other countries. We identified the implicated food for 2 outbreaks (outbreaks 1 and 14) because other countries rapidly published reports in Eurosurveillance Weekly. We alerted other countries to the implicated food for 3 other common-source outbreaks (outbreaks 3 and 5, by using rapid reports in the same publication and outbreak 2 through ProMED Mail) (*11*–*14*).

During these investigations, we attempted to identify other countries that had also received contaminated food. Before the inception of INFOSAN, we relied on diplomatic communications with the exporting country, which were often unsuccessful. In a recent incident in which persons became infected with toxigenic *Vibrio cholerae* after eating raw whitebait (outbreak 13), INFOSAN Emergency made inquiries of the exporting country and confirmed that fish had not been exported to other countries and that no outbreak was observed locally (*15*). During a multicountry outbreak of drug-resistant shigellosis (outbreak 14), INFOSAN Emergency Focal Point at WHO gained the exporting country’s cooperation to trace back the produce to the facility concerned and informed other countries receiving the same batch of produce (*11*) (Appendix Table).

## Conclusions

Although IHR (2005) only came into force in June 2007, we consider that there would have been a basis for reporting 6 (43%) of 14 imported food outbreaks, with 3 of these being part of the same IQF oyster contamination event. Although National IHR Focal Points may decide not to notify or report an outbreak under IHR (2005), it is vital that they publish rapid reports involving imported and exported foods, given the potential of these foods to spread disease internationally, and consult with WHO through INFOSAN Emergency. In this report, we considered only those events that resulted in human illness, but it is important for National IHR Focal Points to consult with the INFOSAN Emergency Contact Point for their country and to consider notifying and/or reporting events in which food is contaminated in the absence of human illness. Serious, unusual, or unexpected events associated with domestic food may also trigger the criteria, even when foods are not exported. Note that under Article 9.2 of IHR (2005), public health risks associated with importation of contaminated goods may be reported to WHO independent of the event’s meeting the Annex II criteria. This stipulation would allow reporting when available information is insufficient to make an adequate assessment under Annex II. We found WHO INFOSAN Emergency complementary to the management of IHR (2005). The role of WHO and other agencies in these events of potential international importance will undoubtedly continue to evolve.

## Supplementary Material

Appendix Table Outbreaks of illness that implicated internationally distributed foods and assessment of questions on the IHR (2005) decision instrument, Australia, 2001-2007*
