# Erratum—Vol. 14, No. 9

**DOI:** 10.3201/eid1412.000999

**Published:** 2008-12

The article Obligations to Report Outbreaks of Foodborne Disease under the International Health Regulations (2005) (M.D. Kirk et al.) contained incorrect figures in the abstract and conclusion. The text stated that 7 (50%) of 14 outbreaks would have required notification to the World Health Organization (WHO). The correct proportion is 6 (43%) of 14 outbreaks that would have required notification to the WHO. The article has been corrected online (www.cdc.gov/eid/content/14/9/1440.htm).

