# Supplementary material for: Obligations to Report Outbreaks of Foodborne Disease under the International Health Regulations (2005)
Source: Emerg Infect Dis. 2008 Sep;14(9):1440–2. doi: 10.3201/eid1409.080468 (PMC2603118; doi:10.3201/eid1409.080468)
Supplement: Appendix Table — Outbreaks of illness that implicated internationally distributed foods and assessment of questions on the IHR (2005) decision instrument, Australia, 2001-2007* [file 08-0468_appT-s1.pdf]

Appendix Table. Outbreaks of illness that implicated internationally distributed foods and assessment of questions on the IHR (2005) decision instrument, Australia, 2001–2007*

| Outbreak no. | Year | Outbreak description | Implicated food | Public health impact serious?† | Event unusual or unexpected for Australia? | Significant risk for international spread? | Significant risk for international travel or trade restrictions? | Should this be reported to IHR? |
|---|---|---|---|---|---|---|---|---|
| 1 | 2001 | 23 cases of *Salmonella* Typhimurium definitive type 104 | Helva from Turkey | No, small outbreak of moderate severity | Yes, food-borne transmission of this multidrug- resistant *Salmonella* strain rare | Yes, food distributed globally; multiple European countries reporting cases | No, increased testing of imported sesame seed products only | Yes |
| 2 | 2001 | 55 cases of *S*. Stanley and *S*. Newport | Dried peanuts from China | No, moderate outbreak of mild illness | No, *Salmonella* serotypes common | Yes, food distributed globally; cases reported in UK and Canada | No, novel food product | No |
| 3 | 2002 | 55 cases of *S*. Montevideo and *S*. Tennessee | Tahini from Egypt | No, moderate outbreak of mild illness | No, *Salmonella* serotypes common | Yes, food distributed globally; cases reported in New Zealand and contaminated product in Canada and UK | No, increased testing of imported sesame seed products only | No |
| 4 | 2002 | 230 cases of suspected norovirus infection | IQF oysters from Japan | No, large outbreak of mild illness | No, suspected viral illness common | No, food vehicle poorly characterized | No, limited information for decision | No |
| 5 | 2003 | 3 cases of *S*. Montevideo | Tahini from Lebanon | No, small outbreak of mild illness | No, *Salmonella* serotypes common | Yes, food distributed globally; see outbreak 3 | No, increased testing of imported sesame seed products only | No |
| 6 | 2003 | 17 cases of suspected norovirus | IQF oysters from Japan | No, small outbreak of mild illness | No, suspected viral illness common | No, food vehicle poorly characterized | No, limited information for decision | No |
| 7 | 2003 | 35 cases of norovirus infection | IQF oysters from Japan | No, small outbreak of mild illness | No, norovirus outbreaks common | Yes, virus detected in imported oysters | Yes, Australia restricted imports | Yes |
| 8 | 2003 | 48 cases of norovirus infection; related to outbreak 7 | IQF oysters from Japan | No, moderate outbreak of mild illness | No, norovirus outbreaks common | Yes, virus detected in imported oysters | Yes, Australia restricted imports | Yes |
| 9 | 2004 | 5 cases of suspected norovirus infection | IQF oysters from Japan | No, small outbreak of mild illness | No, suspected viral illness common | No, food vehicle poorly characterized | No, limited information for decision | No |
| 10 | 2004 | 3 cases of norovirus infection; related to outbreak 7 | IQF oysters from Japan | No, small outbreak of mild illness | No, norovirus outbreaks common | Yes, virus detected in imported oysters | Yes, Australia restricted imports | Yes |
| 11 | 2005 | 5 cases of ciguatera poisoning in a family | Snapper from Fiji | No, small outbreak of; moderate severity | No, ciguatera common | No, domestic importation of fish only | No | No |
| 12 | 2006 | 5 cases of methemoglobinemia in 2 families | Sodium nitrite powder from China | No, small outbreak; moderate severity | Yes, nitrite poisoning rare | Unknown | No | No |
| 13 | 2006 | 3 cases of *Vibrio cholerae* infection | White bait from | No, small outbreak of | Yes, foodborne cholera rare | Yes, fish imported commercially | Yes, countries may have put in place trade | Yes |

| | | | | | | | | |
|---|---|---|---|---|---|---|---|---|
| | | | Indonesia | moderate severity | | | restrictions | |
| 14 | 2007 | 55 cases of *Shigella sonnei* biotype G infection, with related cases in Denmark | Baby corn from Thailand | No, moderate outbreak of moderate severity | Yes, food-borne transmission of multidrug-resistant shigellosis rare | Yes, food distributed to several countries with other countries reporting cases | Yes, countries may have put in place trade restrictions | Yes |

*IHR, International Health Regulations; UK, United Kingdom; IQF, individually quick frozen.

†Although these outbreaks were not considered to have a serious public health impact, the causative agents may cause a serious public health impact in different situations (i.e., response capabilities, population at risk, virulence).
